# Genome-Wide Association Study Reveals the Genetic Basis of Chilling Tolerance in Rice at the Reproductive Stage

**DOI:** 10.3390/plants10081722

**Published:** 2021-08-20

**Authors:** Byeong Yong Jeong, Yoonjung Lee, Yebin Kwon, Jee Hye Kim, Tae-Ho Ham, Soon-Wook Kwon, Joohyun Lee

**Affiliations:** 1 Department of Crop Science, Konkuk University, Seoul 05029, Korea; quddyd1015@konkuk.ac.kr (B.Y.J.); yoon10.lee@gmail.com (Y.L.); ybb0330@naver.com (Y.K.); maxbaragy@naver.com (J.H.K.); lion78@daum.net (T.-H.H.); 2 Department of Plant Bioscience, College of Natural Resources and Life Science, Pusan National University, Miryang 50463, Korea; swkwon@pusan.ac.kr

**Keywords:** GWAS, chilling tolerance, reproductive stage, rice

## Abstract

A genome-wide association study (GWAS) was used to investigate the genetic basis of chilling tolerance in a collection of 117 rice accessions, including 26 Korean landraces and 29 weedy rices, at the reproductive stage. To assess chilling tolerance at the early young microspore stage, plants were treated at 12 °C for 5 days, and tolerance was evaluated using seed set fertility. GWAS, together with principal component analysis and kinship matrix analysis, revealed five quantitative trait loci (QTLs) associated with chilling tolerance on chromosomes 3, 6, and 7. The percentage of phenotypic variation explained by the QTLs was 11–19%. The genomic region underlying the QTL on chromosome 3 overlapped with a previously reported QTL associated with spikelet fertility. Subsequent bioinformatic and haplotype analyses suggested three candidate chilling-tolerance genes within the QTL linkage disequilibrium block: Os03g0305700, encoding a protein similar to peptide chain release factor 2; Os06g0495700, encoding a beta tubulin, autoregulation binding-site-domain-containing protein; and Os07g0137800, encoding a protein kinase, core-domain-containing protein. Further analysis of the detected QTLs and the candidate chilling-tolerance genes will facilitate strategies for developing chilling-tolerant rice cultivars in breeding programs.

## 1. Introduction

Rice is a major crop that provides the staple diet of more than half of the global population, not only in Asia, but also in Australia, the USA, and Europe [1]. Rice is cultivated under various climates, and the effect of weather on the different developmental stages of rice growth has critical impacts on crop yields. The growth, development, and yield of rice are affected by unfavorable environmental conditions, such as drought, extreme temperatures, high salinity, and flooding [2]. Due to its origin in tropical and subtropical regions, rice is particularly sensitive to low nonfreezing temperature stress. In general, the optimum temperature range for rice cultivation is 25–30 °C [3], and damage from low nonfreezing temperature stress can impact the entire cultivation season, from germination to the grain-filling stage. In general, rice germination and seedling growth are adversely affected at temperatures lower than approximately 15 °C, whereas temperatures below approximately 17–20 °C induce chilling stress during the booting, flowering, and grain-filling stages [3].

Chilling stress in vegetative stages, including germination, causes leaf chlorosis, reduction in growth rate and tillering, and low seedling vigor, all of which lead to decreased yields [4,5]. Physiological processes are disrupted under chilling stress as a result of declining photosynthetic efficiency. Declines in chlorophyll content and fluorescence, alongside stomatal closure, contribute to the decline in photosynthetic rates [6,7]. During the reproductive stage, chilling stress reduces panicle branching, spikelet formation, and pollen fertility, resulting in lower grain numbers per panicle and reduced seed-setting rates, both of which are crucial components of yield [8]. The young microspore stage in rice is particularly sensitive to chilling stress [9,10,11], and low nonfreezing temperatures during the reproductive stage lead to chilling-induced pollen sterility as a result of abnormal microspore development [9,12].

Quantitative trait locus (QTL) analysis was used with biparental crosses to investigate the effects of chilling stress at the booting stage in rice [13]. Ctb1 and Ctb2 on chromosome 4 [14] and locus qCTB7 on chromosome 7 [15] were identified using sets of near-isogenic lines. QTLs qCT1, qCT7, and qCT-11 were found using doubled-haploid lines (DHLs) derived from Akihikari and Koshihikari, and qCTB2a and qCTB3 were identified from recombinant inbred lines (RILs) derived from M-202/IR50 crosses [16]. Some of the reported booting QTLs were fine-mapped, including qCTB7, qCTB10-2, and qLTB3 [15]. For qCTB7, 12 candidate genes were identified in the 92 kb target region, including two hydrolase genes (Os07g0575800 and Os07g0577300), two auxin response genes (Os07g0576100 and Os07g0576500), and one ubiquitin-conjugating enzyme E2 gene (Os07g0577400), which could be related to stress responses. [17,18]. For qCTB 10-2, 17 predicted genes were found in the 132.5 kb target region. Four genes (LTP family precursors Os10g11730 and Os10g11750, dehydrogenase (Os10g11810), and expressed protein (Os10g11770)) were proposed as strong candidate genes for chilling tolerance [17]. For qLTB3, an aldehyde oxidase-2-like gene (Os03g0790700) and a gene encoding a DUF family protein (Os03g0806700) were proposed as candidate genes for chilling tolerance [18]. The development of next-generation sequencing (NGS) technologies facilitated the use of genome-wide association studies (GWAS) for dissection of the genetic basis of chilling tolerance in rice. GWAS are particularly useful for identifying QTLs with weak genetic relationships in natural populations [19]. GWAS of chilling stress in rice detected QTLs associated with chilling tolerance during the rice reproductive stage. Shakiba et al. (2017) identified 63 QTLs from GWAS analysis of chilling stress at the germination and reproductive stages in a collection of 421 diverse rice accessions [20]. 

Plants discern chilling stress using a cell membrane receptor, which stimulates signal transduction and downstream activation of chilling-responsive transduction factors and genes to increase chilling tolerance [21]. Production of several hormones, including abscisic acid, salicylic acid, and jasmonic acid, is also induced by chilling-responsive gene activity [22]. As well as activating stress-responsive genes, these hormones elevate chilling tolerance by increasing secondary metabolite synthesis and reactive oxygen species (ROS) scavenging. Moreover, chilling stress leads to calcium signature induction in plants, which activates the inactive form of an inducer of dehydration-responsive elements, C-repeat binding factor (CBF) expression-1. The high expression of osmotically responsive gene1 (hos1), which is a chilling-responsive gene, stimulates activation of the inducer, which then regulates C-binding factors and a range of other transcription factors. These transcription factors regulate several chilling-responsive genes, giving rise to chilling tolerance in plants [23]. Plants also change the fluidity of their plasma membranes by altering the proportions of unsaturated and saturated fatty acids within the membranes. Plants with higher proportions of unsaturated fatty acids in the plasma membrane exhibit higher chilling resistance [24]. 

In this study, NGS-based GWAS mapping was used to identify candidate genes associated with chilling-stress tolerance at the reproductive stage in rice. More than 100 rice germplasm were phenotyped, revealing ~2.3 million high-quality single-nucleotide polymorphisms (SNPs) across all 12 rice chromosomes. 

## 2. Materials and Methods

### 2.1. Plant Materials and Sequencing

A core Korean rice collection, comprising 117 accessions (KRICE-CORE) (Supplementary Table S1), was used in this study [25]. Genomic sequences were obtained using the Illumina HiSeq 2500 Sequencing Systems Platform (Illumina, Inc., San Diego, CA, USA). Average genome coverage was 8×, and filtered reads were aligned to the rice reference genome sequence (IRGSP 1.0). Genomic sequences were filtered as follows, using VCFtools software v0.1.16 [26]: minor allele frequency (MAF) > 5%; max-missing, 0.75; minQ, 30; and minDP, 4. After filtering, 2,369,723 of a possible 6,243,699 SNP sites were retained.

### 2.2. Chilling-Tolerance Evaluation at the Reproductive Stage (Young Microspore Stage)

Individual plants were cultivated in the experimental paddy field at Konkuk University in 15 × 30 cm rows. Plants were cultivated with a conventional rice cultivation method in a natural rice paddy field until the early young microspore stage. Individual plants at the early young microspore stage, with auricle distance of −1 to +1 cm, were transferred to individual 15 cm diameter pots containing paddy field soil. Potted plants were then treated at 12 °C and 70% relative humidity under a 9 h day, with 25,000 Lux/15 h night cycle, for 5 days in a climate-controlled growth chamber (Hanbaek Sci. Bucheon, South Korea). After treatment, plants in a pot were placed in natural climate conditions in the experimental field and cultivated until fully matured. Seeds were collected from mature panicles and used for fertility assessment. Three biological replications were conducted. 

### 2.3. Population Structure and Linkage Disequilibrium Decay Analysis

Population structure was analyzed based on SNPs in 117 rice accessions using ADMIXTURE 1.3.0 software [27]. Cross-validation error was used for estimation of the number of subgroups. Admixture analysis was performed with the ideal number of ancestries (K) of 1–7, and K was estimated using cross-validation error from ADMIXTURE based on tenfold cross-validation. Results were visualized using Pophelper Structure Web APP v1.0.10 (http://pophelper.com, accessed on 8 June 2021) [28]. Plink software [29] was used to generate a principal component analysis (PCA) matrix, and the optimal number (5) of principal components (PCs) was used as a Q-matrix for GWAS correction. The PCA plot was visualized using R v4.0.3. Neighbor-joining (NJ) trees were generated using MEGA X [30], and the result was visualized using Tree of Life (iTOL) v6.1.2 (https://itol.embl.de, accessed on 15 April 2021) [31]. PopLDdecay software [32] was used to calculate linkage disequilibrium (LD). Pairwise LD was calculated for all SNPs and averaged across the whole genome. Chromosomal distance was estimated by LD block, which was half the average of the pairwise correlation coefficient (R^2^).

### 2.4. GWAS

SNPs were filtered using VCFtools [33], with MAF < 5%; max-missing, 0.75; minQ, 30; and minDP, 4, and indels were removed. The fixed and random model Circulating Probability Unification (FarmCPU) [27], implemented using the rMVP FarmCPU package, was employed for GWAS. The kinship matrix was estimated using the mvp.data.kin function. The kinship matrix (K-matrix) was computed to allow FarmCPU to be used with Q-matrix and K-matrix. GWAS results and plots were automatically visualized in rMVP. The Benjamini–Hochberg FDR method was used to control for the multiple testing error rate [34]. Q-values of 0.10 were used for suggestive thresholds, calculated using the following Equation (1):

Suggestive threshold = −*log*10(0.1/Effective marker number) (1)

Finally, the threshold was set as −*log*10(P) = 6.2873 for the identification of associated QTLs. SNP markers located at QTL peaks were designated as lead SNPs. LD decay analysis identified a 276 kb region on either side of the lead SNP as the candidate genome region for gene identification.

### 2.5. Candidate Gene Prediction and Haplotype Analysis

LD decay analysis was used to designate a region containing 276 kb either side of the lead SNP as the candidate region. Annotation of genes within candidate regions were derived from the Rice Annotation Project database (RAP-DB; https://rapdb.dna.affrc.go.jp, accessed on 23 May 2021). After the elimination of missing and heterozygous data, the full SNP marker set was used for haplotype analysis. Haplotypes (in at least five rice accessions) were used for comparative phenotypic analysis. One-way analysis of variance, followed by the least significant difference (LSD) test, was used to compare phenotypic differences between haplotypes.

## 3. Results

### 3.1. Variability in Rice Chilling Tolerance during Meiosis in Anthesis Development

**Figure 1:**
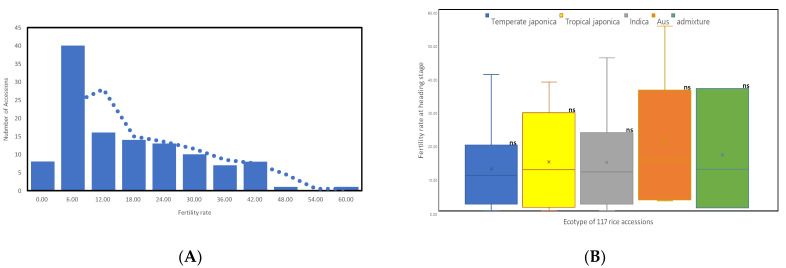
The distribution of fertility for the 117 rice accessions. (**A**) Histogram of the spikelet fertility. The dotted line is the moving average; (**B**) ns—right shoulder represents non-significant different levels of statistical Duncan’s test at ᾳ = 0.05.

A total of 117 Korean rice accessions (KRICE-CORE) were used, comprising 26 Korean landraces, 29 weedy rice accessions collected in Korea, 50 rice germplasms from outside Korea, and 12 Korean rice varieties. In a previous study, these 117 accessions were categorized into five subgroups using 169 simple sequence repeat (SSR) marker genotypes: *tropical japonica*, *temperate japonica*, *indica*, *aus*, and *admixed* [35]. All of the 117 accessions showed normal fertility above 90% under the normal cultivated condition in the natural paddy field in Korea. All 26 Korean landraces and 29 of the weedy rice accessions are native to the Korean peninsula. Although the Korean peninsula is located in the temperate climate region, previous genotype analysis assigned Korean landraces and weedy rice accessions to four ecotype groups on the basis of their genetic relationships: *aus*, *indica*, *tropical japonica*, and *temperature japonica* [36,37]. Chilling tolerance at the early young microspore stage was evaluated with spikelet fertility. The distribution of chilling tolerance was skewed, with 57 accessions having <10% spikelet fertility (Figure 1A). The average spikelet fertility of the 117 accessions was 26.82%. RWG-023 (Hanyangjo; Korean landrace) was the most chilling-tolerant accession, with 55% spikelet fertility. RWG-023 was grouped into the *Aus* ecotype, alongside RWG-122 (ChungdoHwayang12; Korean weedy rice), which had 45% spikelet fertility. RWG-021 (Saducho; Korean landrace) exhibited 41% spikelet fertility and was grouped into the *indica* ecotype. No statistical differences were detected in chilling tolerance between the five ecotype groups (Figure 1B).

### 3.2. Population Structure and LD Decay

**Figure 2:**
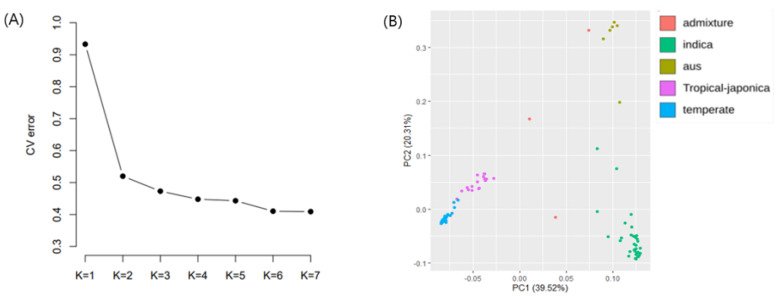

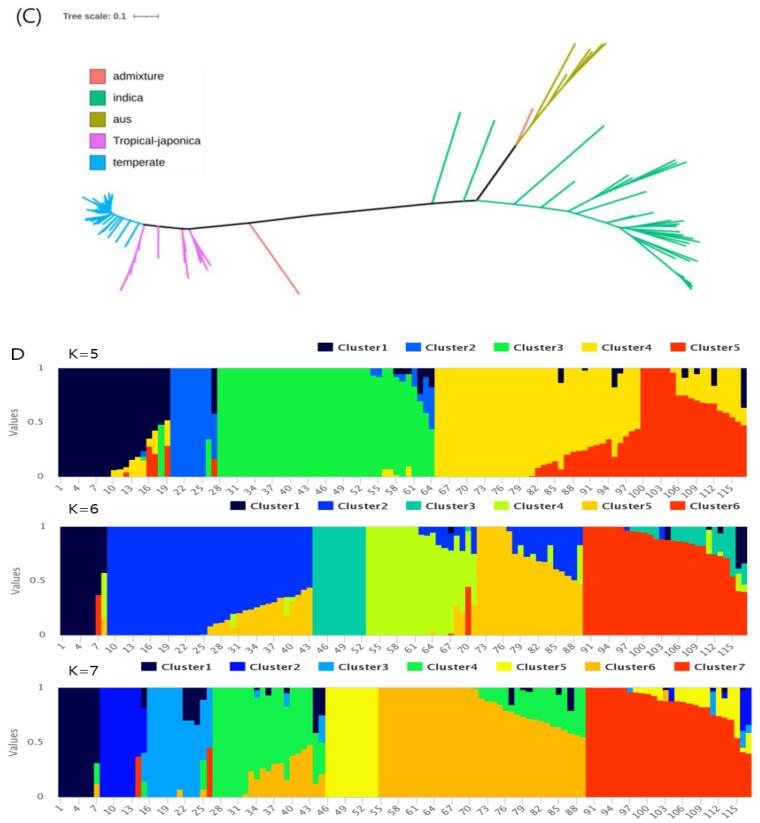
Population structure analysis based on 117 rice accessions. (**A**) Cross-validation error score of each K value. The best K value (K = 6) was selected for population structure analysis. (**B**) Plot for principal component analysis (PCA). Orange, olive green, green, blue, and purple indicated admixture, aus, indica, temperate japonica, tropical japonica rice, respectively. (**C**) Neighbor-joining tree (NJ tree) of rice accessions. The colors represent the same as that used in PCA analysis. (**D**) Plot for population structure analysis at K = 5, 6, 7.

The genetic structure of 117 rice accessions was assessed using ADMIXTURE analyses (Figure 2D). Cross-validation error values from ADMIXTURE analysis showed similar patterns with K values from 4–7. Although the CV error of K = 7 had the lowest value, clustering with K = 7 appeared noisy compared with clustering at K = 5 or K = 6 (Figure 2A). Although previous research suggested five groups [37], in this study, K = 6 provided clearer clustering than K = 5. At K = 6, the accessions were divided into six groups that were mostly distinguished by their subspecies (Figure 2D). 

Temperate japonica accessions were divided between cluster 2 and cluster 5. Tropical japonica was dominant in cluster 4, aus was dominant in cluster 1, and indica accessions were divided between cluster 3 and cluster 6. PCA was performed using 962,055 SNPs. The five previously described subgroups were well separated by the first two PCs (Figure 2B), with PC1 and PC2 explaining 39.52% and 20.31% of the total variation in population structure, respectively.

Accessions grouped as *temperate japonica*, *tropical japonica*, *indica*, and *aus* formed significantly distinct clusters, whereas the admixed accessions exhibited ambiguous separation. In addition, an NJ tree constructed based on Nei’s genetic distance revealed five clusters (Figure 2C), consistent with the PCA separation among groups. Most accessions were clearly separated, with admixed accessions dispersed among the different clusters. PCA and NJ tree PCs (Figure 2B,C) clearly differentiated the indica and japonica subpopulations. Temperate japonica and tropical japonica were also distinguished from one another, but were closely related. Aus was distinguished from both indica and japonica, but was closely related to indica. Decay of LD along physical distance was computed for the full panel of rice accessions. The *r*^2^ value declined with increasing physical distance. The threshold value for candidate regions was determined as half of the maximal *r*^2^ value (0.276), producing a candidate genomic region of 276 kb. 

### 3.3. GWAS of Chilling-Stress Tolerance during Anthesis Development 

**Figure 3:**
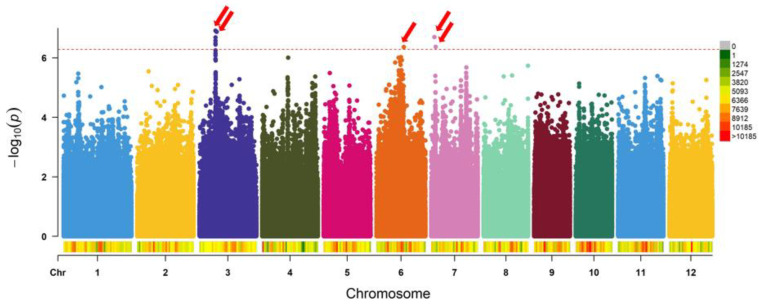
Manhattan plots for plant fertility after low temperature (12 °C for 5 days) treatment for 117 rice accessions. SNP density is indicated by the color scale on the bar next to the *x*-axis (scale given in right upper corner). It indicates the $-log_{10}\;\left(P\right)$ value on the *y*-axis and the SNP position of each chromosome on the *x*-axis. The horizontal blue line indicates thresholds ($-log_{10}\;\left(P\right)$ = 6.2873).

Manhattan plots of SNP markers significantly associated with chilling tolerance at the seedling stage are presented in Figure 3. SNPs with a −*log*10(P) score of 6.2873 were considered to be significantly associated with chilling tolerance. Nine significant SNPs associated with chilling tolerance were detected. Significant SNPs within a 552 kb range were considered as one association locus and, in total, five QTLs were mapped, on chromosomes 3, 6, and 7 (Table 1). The percentage of phenotypic variation explained by QTLs was 11–19%, where qCTR7-1 showed the largest explained percentage of 19%. LD block size was 552 kb surrounding the lead SNP, and the genomic segments corresponding to these five QTLs were defined and then compared with those of previously reported QTLs (Table 1). Overlaps were found between the loci detected by GWAS in this study and previously reported QTLs linked to various agronomic traits. 

However, none of the previously reported QTLs were associated with chilling tolerance in the reproductive stage. QTLs associated with stress, fertility, or spikelet morphology were identified, and these traits may be indirectly associated with chilling tolerance during the reproductive stage. Comparative analysis revealed that qCTR3-1 on chromosome 3 overlapped with the AQFT003 QTL, which was associated with osmotic adjustment capacity [38]. On chromosome 3, qCTR3-2 overlapped with the AQCU106 QTL, which was associated with spikelet fertility [39]. On chromosome 6, qCTR6 overlapped with the CQAR19 QTL, which was associated with spikelet density [40]. On chromosome 7, qCTR7-1 overlapped with the CQAV4 QTL, which was also associated with osmotic adjustment capacity [41]. Moreover, on chromosome 7, qCTR7-2 overlapped with the AQEM004 QTL, which was associated with salt sensitivity [42]. 

### 3.4. Haplotype Analysis to Identify Chilling Tolerance Candidate Genes 

**Figure 4:**
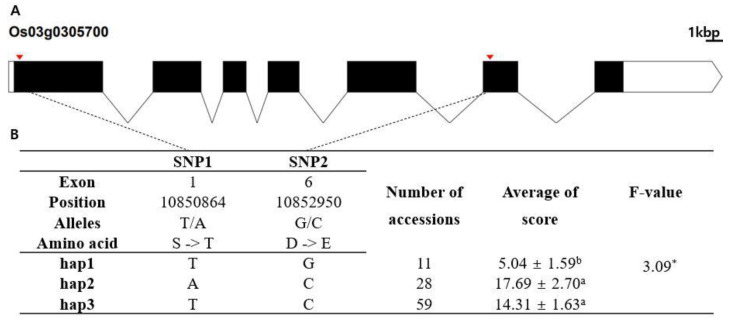
Haplotype analysis result of Os03g0305700. (**A**) Gene structure and SNP positions on Os03g0305700. Black box and line indicate exons and introns, respectively. Red marks indicate SNPs. (**B**) Significant haplotypes by LSD test at α = 0.05. Letters a and b represent different levels of LSD test. *: significant level at the 5% level.

**Figure 5:**
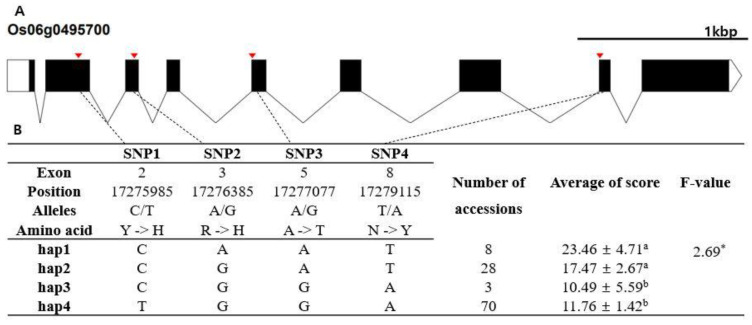
Haplotype analysis result of Os06g0495700. (**A**) Gene structure and SNP positions on Os06g0495700. Black box and line indicate exons and introns, respectively. Red marks indicate SNPs. (**B**) Significant haplotypes by LSD test at α = 0.05. Letters a and b represent different levels of LSD test. *: significant level at the 5% level.

**Figure 6:**
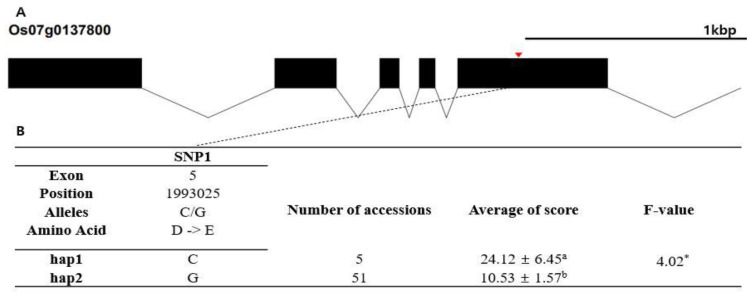
Haplotype analysis result of Os07g0137800. (**A**) Gene structure and SNP positions on Os07g0137800. Black box and line indicate exons and introns, respectively. Red marks indicate SNPs. (**B**) Significant haplotypes by LSD test at α = 0.05. Letters a and b represent different levels of LSD test. *: significant level at the 5% level.

In silico analysis was conducted to identify candidate genes responsible for chilling tolerance. The RAP-DB database (IRGSP 1.0) was used to identify annotated genes in the 552 kb regions encompassing the five lead SNPs. A total of 83 genes were found in the encompassing region of qCTR3-1, 81 genes for qCTR3-2, 41 genes for qCTR6, 70 genes for qCTR7-1, and 53 genes for qCTR7-2. Hypothetical protein genes were not used for subsequent haplotype analysis and, in total, 213 of the 328 identified genes in the five QTL regions were subjected to haplotype analysis. Phenotypic comparison was conducted for haplotypes containing at least five rice accessions. Finally, 26 candidate genes showing statistically significant differences between the haplotypes were detected (Supplementary Table S2). Based on the phenotypic differences between haplotypes and the functional annotation of genes, three candidate genes were selected for further analysis. Haplotype analyses of the three candidate genes are presented in Figure 4, Figure 5 and Figure 6. Heterozygous SNPs and SNPs with missing data were not included in the analysis. SNPs in exons were used for haplotype and haplotype variation analysis. Os03g0305700 (similar to peptide chain release factor 2) contained two SNPs within exons (Figure 4A). The two SNPs were nonsynonymous and located in the coding region (T→A, Chr3_10850864, S→T substitution; G→C, Chr3_10852950, D→E substitution) of the dienelactone hydrolase family domain. The two SNPs formed three haplotypes, Hap1, Hap2, and Hap3 (Figure 4B). The mean chilling tolerance of Hap2 (score, 17.6891; 28 accessions) was higher than that of Hap1 (score, 5.03673; 11 accessions). Os06g0495700 (beta tubulin, autoregulation binding-site-domain-containing protein) contained four nonsynonymous SNPs in exons (C→T, Chr6_17275985, Y→H substitution; A→G, Chr6_17276385, R→H substitution, A→G, Chr6_17277077, A→T substitution; A→T, Chr6_17279115, N→Y substitution), forming four haplotypes (Hap1, Hap2, Hap3, and Hap4) (Figure 4). The mean chilling-tolerance score of Hap1 (23.4644, eight accessions) was higher than that of Hap3 (10.4898, three accessions) and that of Hap4 (11.7632, 70 accessions). Os07g0137800 (protein kinase, core-domain-containing protein) contained a single nonsynonymous SNP (C→G, Chr7_1993025, D→E substitution) that formed two haplotypes (Hap1 and Hap2) (Figure 5B). The fertility of Hap1 differed significantly from that of Hap2.

## 4. Discussion

Chilling tolerance of rice during the reproductive stage is crucial for stable yield production. Chilling temperature exposure during this highly sensitive stage prevents development of normal pollen, resulting in significant yield loss. Chilling-tolerance distribution in the 117 rice accessions was skewed, with 47.86% of the accessions exhibiting <10% spikelet fertility. This reflected the high sensitivity of the early young microspore stage to chilling exposure. The temperate japonica rice ecotype is generally thought to be more chilling tolerant than the aus and indica ecotypes. However, no differences in chilling tolerance between the ecotypes were observed here. Moreover, accession RWG-023, which grouped as aus, was the most chilling-tolerant accession, with 55% spikelet fertility. RWG-122, which also grouped as aus, exhibited 45% spikelet fertility, while RWG-021, exhibiting 41% spikelet fertility, grouped as indica. Although these chilling-tolerant accessions were grouped as aus or indica, generally regarded as chilling-susceptible ecotypes, these lines belonged to the native germplasm of the Korean peninsula, which has a temperate climate. RWG-021 and RWG-023 are Korean landraces, and RWG-122 is a Korean weedy rice. The unexpected reproductive chilling tolerance of these accessions suggests that the chilling-tolerance trait may not be associated with ecotype differentiation in the Korean peninsula. Although the dissociation between reproductive chilling tolerance and ecotype was not clearly elucidated in our study, these germplasms may be beneficial for introducing the chilling-tolerance trait into aus and indica varieties as a genetically closely related donor parent.

Previously reported QTLs overlapping the physical positions of the newly identified QTLs were investigated. Three stress-associated QTLs, one spikelet-fertility-associated QTL, and one panicle-associated QTL were found. The qCTR3-1 QTL overlapped the AQFT003 QTL, which was associated with osmotic adjustment capacity. AQFT003 was detected by QTL analysis of drought tolerance at the reproductive stage in DHLs derived from a cross between upland japonica (CT9993) and indica cultivars (IR62266) [38]. The qCTR3-2 QTL overlapped the AQCU106 QTL, which was associated with spikelet fertility. AQCU106 was identified in an RIL population derived from a cross between Lemont (japonica) and Teqing (indica) varieties [39]. The qCTR6 QTL overlapped the CQAR19 QTL, which was associated with spikelet density. CQAR19 was detected by QTL analysis of an F2 mapping population derived from indica Zhaiyeqing 8 and japonica Jingxi 17 [40]. The qCTR7-1 QTL overlapped the CQAV4 QTL, which was associated with osmotic adjustment capacity. CQAV4 was detected by QTL analysis of drought tolerance at the seedling stage, with RILs derived from a cross between an upland japonica cultivar (Moroberekan) and an indica cultivar (Co39) [41]. The qCTR7-2 QTL overlapped the AQEM004 QTL, which was associated with salt sensitivity. AQEM004 was detected by QTL analysis of F_2_ and an equivalent F_3_ population developed from a cross between a high-salt-tolerant indica variety, Nona Bokra, and a salt-susceptible elite japonica variety, Koshihikari, [42].

The rice genome database [43]. was used to identify 328 candidate genes in the LD blocks of the detected QTLs. After haplotype analysis and functional annotation of the genes, three candidate genes were selected for further consideration: Os03g0305700, Os06g0495700, and Os07g0138400. Expression profiling data for the three candidate genes were obtained from the Transcriptome Encyclopedia of Rice (TENOR) database (http://tenor.dna.affrc.go.jp/, accessed on 20 June 2021) [44]. Os03g0305700 is predicted to encode a peptide chain release factor 2 family protein. According to TENOR, expression of Os03g0305700 increased in seedlings under chilling conditions (Supplementary Figure S1). A release factor is a protein that allows for the termination of translation by recognizing the termination codon or stop codon in an mRNA sequence. Although it is widely believed that translation termination is a highly conserved process in eukaryotes, the role of translation termination in plant development is largely unknown. Eukaryotic release factor 1–2 is involved in hypersensitive responses to glucose and phytohormones during germination and early seedling development [45]. Os06g0495700 is predicted to encode a beta tubulin. According to TENOR, expression of Os06g0495700 increased under drought and flood conditions (Supplementary Figure S1). Beta tubulin microtubules have important roles in many cellular processes, including cell division and cell elongation in plants. The organization and stability of plant microtubules are affected by environmental stresses, such as dehydration, high salinity, low nonfreezing temperature, and aluminum exposure [46]. Post-meiotic radial microtubule arrays in Arabidopsis male gametes undergo depolymerization in response to chilling stress [47]. In Arabidopsis, chilling stress alters the formation of radial microtubule arrays at telophase II and consequently leads to defects in meiosis during pollen development [48]. Expression of beta tubulin also decreased in Arabidopsis leaves upon exposure to low nonfreezing temperatures [48]. Os07g0137800 is predicted to encode a protein kinase. According to TENOR, Os07g0137800 expression increased under high salinity, low phosphate, and high phosphate conditions (supplementary Figure S1). In addition, Ling et al. (2015) used qPCR analysis to confirm that Os07g0137800 was preferentially expressed in the mature anther [49]. Protein kinases regulate the biological activity of proteins by phosphorylation of specific amino acids, with ATP as the source of phosphate. Protein kinases are involved in various plant responses to environmental stresses such as drought, high salinity, chilling, and pathogen attack [50]. Increased expression of OsCDPK7, a calcium-dependent protein kinase, was detected upon exposure to chilling and salt stresses, and overexpression of OsCDPK7 in transgenic rice increased tolerance to chilling and salt stress during the vegetative stage, suggesting that OsCDPK7 acted as a positive regulator during the tolerance response to both stresses in rice [51]. Based on the function of protein kinase, the expression pattern of Os07g0137800, and the previously reported role of calcium-dependent protein kinase in chilling tolerance, Os07g0137800 is a strong candidate for further evaluation for its possible role in chilling tolerance during pollen development. 

## Figures and Tables

**Table 1:** QTLs detected in GWAS and previously reported QTLs.

QTL	Chr	Lead SNP	−*log*10(P)	Reported QTL		Reference
				QTL Accession ID	Related Trait	
qCTR3-1	3	10,331,835	6.91	AQFT003	Osmotic adjustment capacity	Jonaliza C. et al., 2004 [38]
qCTR3-2	3	11,063,641	6.87	AQCU106	Spikelet fertility	Mei H.W. et al., 2003 [39]
qCTR6	6	17,362,869	6.36	CQAR19	Spikelet density	Xu Y.B. et al., 1995 [40]
qCTR7-1	7	1,976,872	6.70	CQAV4	Osmotic adjustment capacity	Lilley J.M. et al., 1996 [41]
qCTR7-2	7	2,881,574	6.37	AQEM004	Salt sensitivity	Lin H.X. et al., 2004 [42]

## Supplementary Materials

The following are available online at https://www.mdpi.com/article/10.3390/plants10081722/s1, Figure S1: Expression profiles in rice seedling under the various environmental conditions. Table S1: A core Korean rice collection, 117 accessions; Table S2: The 26 candidate genes.
