# Supplementary material for: Genome-Wide Association Study Reveals the Genetic Basis of Chilling Tolerance in Rice at the Reproductive Stage
Source: Plants (Basel). 2021 Aug 20;10(8):1722. doi: 10.3390/plants10081722 (PMC8398597; doi:10.3390/plants10081722)
Supplement: Supplementary file 1 [file plants-10-01722-s001.zip › supplymentary figure 1.pdf]

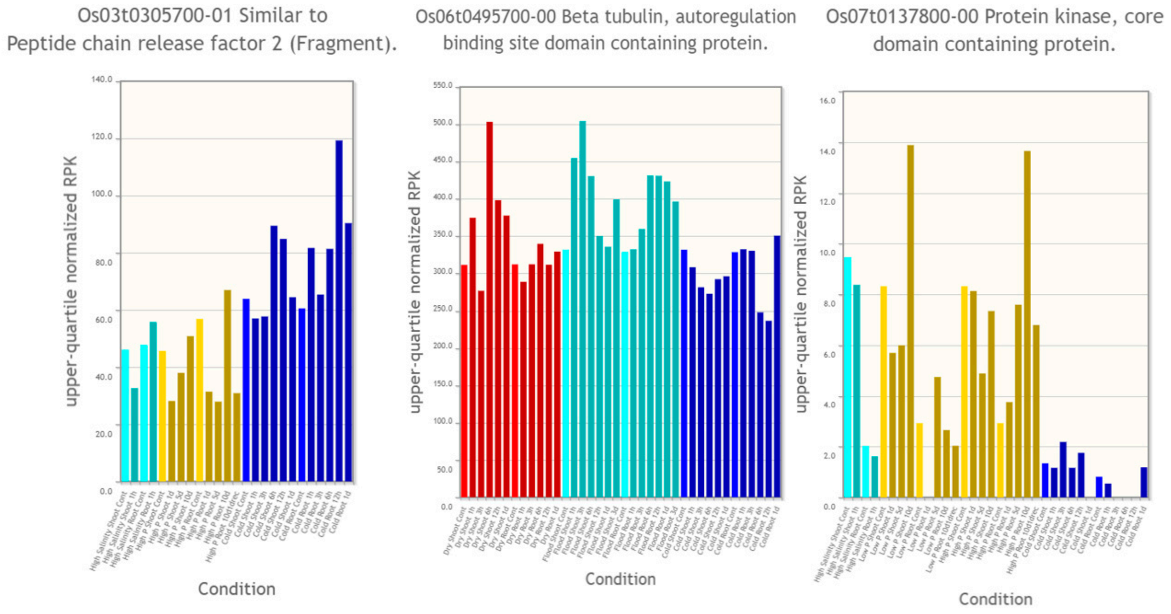

Supplementary figure S1. Expression profiles in rice seedling under the various environmental conditions.
