# Supplementary material for: Genome-Wide Association Study Reveals the Genetic Basis of Chilling Tolerance in Rice at the Reproductive Stage
Source: Plants (Basel). 2021 Aug 20;10(8):1722. doi: 10.3390/plants10081722 (PMC8398597; doi:10.3390/plants10081722)
Supplement: Supplementary file 1 [file plants-10-01722-s001.zip › Supplymentary table 1.pdf]

Supplymentary table 1. A core Korean rice collection, 117 accessions.

Resource	Name	Ecotype	Classification	Fertility
RWG-023	Hanyangjo	Aus	Landrace	55.10204
RWG-122	ChungdoHwayang12	Indica	Weedy	45.63267
RWG-021	Saducho	Indica	Landrace	41.25879
RWG-079	Gou 405	Temperate japonica	Intro	40.7274
RWG-102	Sando	Tropical japonica	Landrace	38.44957
RWG-035	BELLARDONE	Temperate japonica	Intro	38.09754
RWG-098	NeulByeo	Tropical japonica	Landrace	38.0609
RWG-067	XI GUA BAI	Indica	Intro	36.732
RWG-031	Mongdonjaera	admixture	Landrace	36.50496
RWG-018	Sando	Tropical japonica	Landrace	36.09023
RWG-048	Yangmyeon	Indica	Intro	34.62879
RWG-050	Pyeongbuk3	Temperate japonica	Intro	34.53018
RWG-059	DoanGuangHwaLue	Temperate japonica	Intro	33.79592
RWG-004	SUWON 301	Temperate japonica	Bred	32.58166
RWG-078	Hwaseong-5	Temperate japonica	Weedy	31.12169
RWG-015	Muando	Temperate japonica	Landrace	31.03081
RWG-003	SUWEON 255	Indica	Bred	30.61265
RWG-066	URASAN	Tropical japonica	Intro	29.96484
RWG-008	TCHAMPA	Aus	Intro	29.6875
RWG-125	Jangseong 1	Indica	Weedy	29.09408
RWG-090	Jejubukjeju-2002-115	Tropical japonica	Weedy	29.01577
RWG-118	TSONG-GAN-SHUN	Indica	Intro	28.50242
RWG-028	Yuljojo	Temperate japonica	Landrace	27.12121
RWG-060	WuCyue	Indica	Intro	26.74715
RWG-046	VICTORIA F.A	Temperate japonica	Intro	25.95476
RWG-135	Milyang88	Temperate japonica	Bred	24.32664
RWG-111	XaoBaiMangSueDao	Temperate japonica	Intro	24.01458
RWG-085	MILYANG 50	Indica	Bred	23.54316
RWG-086	YeongpungByeo	Indica	Bred	23.00765
RWG-022	SEON	Indica	Landrace	22.37643
RWG-081	AKAINE	Tropical japonica	Intro	21.02273
RWG-070	QUA 77 WUAN-DAU	Indica	Intro	20.93916
RWG-053	KAGI	Aus	Intro	20.82313
RWG-137	IR40	Indica	Intro	20.70115
RWG-099	JwiippariByeo	Temperate japonica	Landrace	20.17702
RWG-097	Seorianjeunbaengi	Temperate japonica	Landrace	19.71935
RWG-094	Jotajo	Temperate japonica	Landrace	19.49459
RWG-069	KENG CHI JU	Temperate japonica	Intro	19.10331
RWG-119	SAN-LI-SHUN	Indica	Intro	18.45199
RWG-036	CHIEM CHANK	Indica	Intro	18.23778
RWG-002	SUWEON 159	Temperate japonica	Bred	17.93188

RWG-033	ANBAW C7	Temperate japonica	Intro	17.11806
RWG-104	Naengjo	Temperate japonica	Landrace	15.41527
RWG-121	Chungdo23	Indica	Weedy	14.95007
RWG-016	Dadajo	Temperate japonica	Landrace	14.84905
RWG-026	JANMOCHAL	Temperate japonica	Landrace	14.81522
RWG-065	SSALBYEO 22	Temperate japonica	Weedy	14.29224
RWG-072	HSIANG-HA-TSAN	Indica	Intro	14.22287
RWG-012	Jejubukjeju-2002-420	Temperate japonica	Weedy	13.99593
RWG-045	TUN SART	Tropical japonica	Intro	13.73697
RWG-032	AGBEDE	Tropical japonica	Intro	13.02982
RWG-056	WAIKYAKUSHI	Indica	Intro	12.60006
RWG-042	MUSHKAN	admixture	Intro	12.36595
RWG-089	MOROBEREKAN	Tropical japonica	Intro	12.23355
RWG-096	DongoByeo	Temperate japonica	Landrace	12
RWG-040	MAGNOLIA	Tropical japonica	Intro	11.99478
RWG-095	PocheonJangmangMebyeo	Temperate japonica	Landrace	11.91292
RWG-009	Jejubukjeju-2002-9	Temperate japonica	Weedy	11.85542
RWG-093	Huindadak	Temperate japonica	Landrace	11.76471
RWG-123	ChungdoHwayang14	Indica	Weedy	11.60586
RWG-076	Danyang-7	Temperate japonica	Weedy	10.96982
RWG-112	Dijiaowujian	Indica	Intro	9.996202
RWG-005	Suweon 347	Indica	Bred	9.98785
RWG-011	Jejubukjeju-2002-340	Temperate japonica	Weedy	9.864573
RWG-037	DHARIAL	Aus	Intro	9.389671
RWG-131	Suhyeonghando	Indica	Intro	9.31601
RWG-084	Syalebyeo-163-1-B	Temperate japonica	Weedy	8.571064
RWG-075	Golyeong-6	Temperate japonica	Weedy	7.647731
RWG-091	Jejubukjeju-2002-55	Temperate japonica	Weedy	7.519715
RWG-019	Batnarak	Tropical japonica	Landrace	6.666667
RWG-100	Jeongjonghwa	Temperate japonica	Landrace	5.99882
RWG-014	kanghwasujip-16	Temperate japonica	Weedy	5.619413
RWG-132	Xiaozaohuang	Tropical japonica	Intro	5.432641
RWG-136	Dudo	Temperate japonica	Landrace	5.024337
RWG-126	Suncheon 5	Indica	Weedy	4.964539
RWG-087	IRI 336	Temperate japonica	Bred	4.671101
RWG-001	NAMSEON 52	Temperate japonica	Bred	4.451981
RWG-047	BaiCyueHwaLue	Indica	Intro	4.146844
RWG-074	Golyeong-2	Temperate japonica	Weedy	3.786948
RWG-113	SPIN MERE	Aus	Intro	3.333333
RWG-106	BRITISH HONDURAS CREALE	Tropical japonica	Intro	3.050221
RWG-041	MALA	Indica	Intro	2.884703
RWG-108	SAHAK	Aus	Intro	2.791839
RWG-117	SUNG PAN TAO	Temperate japonica	Intro	2.777778

RWG-092	Jejubukjeju-2002-561	Temperate japonica	Weedy	2.563194
RWG-114	NORIN 22	Temperate japonica	Intro	2.301587
RWG-116	NIAN CHI SHI	Indica	Intro	1.958422
RWG-082	Syarebyeo-61-1-B	Temperate japonica	Weedy	1.929005
RWG-124	Seongju3	Indica	Weedy	1.903798
RWG-109	TAICHUNG-WOO-TSAN	Indica	Intro	1.86267
RWG-128	Guechang15	Temperate japonica	Weedy	1.81686
RWG-064	HAWM SUPAN	Indica	Intro	1.785714
RWG-027	Pyodo	Temperate japonica	Landrace	1.709402
RWG-010	Jejubukjeju-2002-171	Indica	Weedy	1.574803
RWG-088	MihyangByeo	Temperate japonica	Bred	1.511821
RWG-083	Syalebyeo-94-1-B	Temperate japonica	Weedy	1.504024
RWG-073	Cheongdo-donggok-4	Indica	Weedy	1.449275
RWG-006	CT9993-5-10-1-M	Indica	Intro	1.166858
RWG-030	BAEKGOGNA	Indica	Landrace	1.071429
RWG-068	YUNG YUEN CHUEN ZIM	Indica	Intro	1.020408
RWG-058	NEWREX	Tropical japonica	Intro	0.941467
RWG-051	YUPUL	Tropical japonica	Intro	0.939166
RWG-129	Danyang9	Temperate japonica	Weedy	0.884956
RWG-127	Daegu damti 6-2	Temperate japonica	Weedy	0.864109
RWG-105	BIKOM	admixture	Intro	0.8
RWG-017	OKCHEONG	Temperate japonica	Landrace	0.75188
RWG-134	NAMSEON 126	Temperate japonica	Bred	0.724638
RWG-024	Inbujido	Temperate japonica	Landrace	0.657895
RWG-057	UPLAND	Tropical japonica	Intro	0.60241
RWG-130	Chungsongaengmi4	Temperate japonica	Weedy	0.45045
RWG-013	Jeju collection	Indica	Weedy	0
RWG-044	RED RICE	Indica	Intro	0
RWG-054	HATADANI	Indica	Intro	0
RWG-025	Beobpanhwa	Temperate japonica	Landrace	0
RWG-077	Danyang-38	Temperate japonica	Weedy	0
RWG-133	NAMSEON 34	Temperate japonica	Bred	0
RWG-055	TAI MOCHITO	Tropical japonica	Intro	0
