# Supplementary material for: Genome-Wide Association Study Reveals the Genetic Basis of Chilling Tolerance in Rice at the Reproductive Stage
Source: Plants (Basel). 2021 Aug 20;10(8):1722. doi: 10.3390/plants10081722 (PMC8398597; doi:10.3390/plants10081722)
Supplement: Supplementary file 1 [file plants-10-01722-s001.zip › supplymentary table 2.pdf]

Candidate gene	p-value	haplotype	accession	mean
Os03g0293600	0.008286	hap1	28	19.83602
		hap2	78	12.1916
Os03g0299800	0.046701	hap1	77	13.25845
		hap2	7	3.632587
Os03g0305100	0.027405	hap1	11	5.895067
		hap2	105	14.9299
Os03g0305700	0.021188	hap1	11	5.036729
		hap2	28	17.68908
		hap3	59	14.30581
Os03g0308100	0.038766	hap1	67	14.98514
		hap2	16	7.53365
Os03g0311600	0.035177	hap1	8	20.15117
		hap2	8	3.534199
		hap3	55	14.4928
Os06g0493801	0.013151	hap1	2	3.062586
		hap2	8	22.92597
		hap3	49	11.19535
		hap4	3	5.501724
		hap5	11	20.55742
Os06g0494000	0.018717	hap1	44	17.56909
		hap2	73	11.77322
Os06g0494100	0.005486	hap1	2	3.062586
		hap2	40	19.12631
		hap3	74	11.62493
Os06g0494400	0.022423	hap1	2	3.062586
		hap2	5	11.65349
		hap3	10	17.85106
		hap4	2	6.182975
		hap5	48	10.30353
		hap6	6	26.90764
Os06g0495100	0.0000000002	hap1	61	12.01783
		hap2	28	27.3125
		hap3	8	9.694598
		hap4	2	2.967462
		hap5	10	1.356132
		hap6	3	0.000001
Os06g0495500	0.044923	hap1	3	5.501724
		hap2	15	15.01718
		hap3	55	10.07418
		hap4	7	21.92041

Os06g0495700	0.033277	hap1	8	23.46438
		hap2	28	17.46683
		hap3	3	10.48975
		hap4	70	11.76322
Os06g0497500	0.026556	hap1	11	19.25928
		hap2	72	11.66356
		hap3	19	19.2096
Os06g0498500	0.02358	hap1	8	20.42438
		hap2	2	3.062586
		hap3	29	18.51895
		hap4	72	11.45986
Os06g0498900	0.017959	hap1	2	3.062586
		hap2	74	11.71374
		hap3	5	26.2962
Os06g0499000	0.015496	hap1	10	22.61646
		hap2	33	16.57198
		hap3	74	11.62493
Os06g0499550	0.167239	hap1	8	20.0137
		hap2	6	12.36236
		hap3	23	16.67147
		hap4	70	11.51564
Os06g0500100	0.034982	hap1	101	12.83605
		hap2	6	26.5284
		hap3	6	12.36236
Os06g0500300	0.014102	hap1	76	11.81034
		hap2	41	17.94388
Os06g0500700	0.003817	hap1	73	12.03401
		hap2	22	16.92117
		hap3	6	29.03965
Os07g0137800	0.014819	hap1	5	24.11694
		hap2	51	10.53287
Os07g0150200	0.008944	hap1	6	4.151038
		hap2	86	12.89765
		hap3	22	20.33397
Os07g0150500	0.017033	hap1	28	19.00924
		hap2	86	12.2751
Os07g0150700	0.01128	hap1	29	19.16558
		hap2	87	12.14418
Os07g0151200	0.015088	hap1	7	13.49861
		hap2	16	22.33665
		hap3	70	12.02965

Supplymentary table 2. The 26 candidate genes.
